# Supplementary material for: Dynamic blood oxygen indices in mechanically ventilated COVID-19 patients with acute hypoxic respiratory failure: A cohort study
Source: PLoS One. 2022 Jun 10;17(6):e0269471. doi: 10.1371/journal.pone.0269471 (PMC9187096; doi:10.1371/journal.pone.0269471)
Supplement: Attachment — Submitted filename: Response to reviewers comments.docx [file pone.0269471.s001.docx]

**To**

**Editorial Team PLOS One**

Acute Perioperative and Critical Care Group

Southampton NIHR Biomedical Centre

CE93

Mailpoint 24

E-Level, Centre Block

Southampton General Hospital

Tremona Road

Shirley

Southampton

SO16 6YD

|  | Tel: 07903943418  Fax: 023 8079 5378 |
| --- | --- |

20 April 2022

Thank you for the valuable comments. Please see our response to the reviewer’s and editorial comments.

**Reviewer #1:**

Your work is applaudable. Please find below some comments and suggestions:

1. "Due to the nature of this study, the need for individual informed patient consent was waived." Can the authors provide further explanation as to why the waiver is applicable to this particular study?

***Response:*** *Thank you for your comment. We have now modified this sentence to clarify this further as “due to the retrospective and observational nature of the study and there were no identifiable patient’s source data, the need for individual informed patient consent was waived. The data analysed were already routinely collected and electronically stored as part of clinical care”*

2. "We did not exclude any patients based on the presence of co-morbidities that may have contributed to their death or those enrolled in other clinical trials." Can the authors provide further explanation as to why such patients were not within the frames of an exclusion criteria?

***Response****: Thank you. We have now modified this sentence to clarify this statement further. “As a pragmatic retrospective study without intervention, we evaluated the merit of various oxygen indices under a real-life, generalised intensive care setting which may be applicable to routine practice. We therefore did not exclude any patients based on the presence of comorbidities that may have contributed to their death or those enrolled in other clinical trials”.*

3. Can the authors provide their limitation in a separately headed segment? Or present it separately from the first statement of the paragraph that highlights limitation?

***Response****: We have now presented the limitations as a separate paragraph.*

4. Can the authors provide statements in their conclusion that highlight the importance of their findings in terms of shaping clinical practice or global/public health practices?

*Response: Thank you. While this is an important clinical finding that oxygen utilisation may be compromised in severe fatal COVID-19 patients, this warrants further clinical and scientific explorations to shape any future clinical practice (i.e., measure to improve oxygen utilisation, is not straight forward). This a pragmatic observational study suggests that improving oxygen content alone may not be adequate. Any additional comments on change in clinical practice would be exploratory.*

**Reviewer #2:**

This retrospective cohort study evaluated relationship between three oxygen indices and clinical outcomes in mechanically ventilated COVID-19 patients. Although concept of the study is reasonable and the manuscript is well written, methods have serious problem.

Major points:
1) As authors mentioned in a limitation part (P22, L364), the results of this study are affected by serious sampling bias, which has bearings on the fundamental point of the study design. Including all blood samples into the analysis cannot be reasonable, since more samples from sicker patients must lead to patently false conclusion. I totally agree with authors opinion that oxygen extraction rate is associated with mortality of COVID-19 patients, but I cannot agree this study is technically sound and appropriate, as long as they use quite biased samples. Since this study included 181 patients, number of blood gas samples must be multiples of 181.

*Response: Thank you for your comment. This was a pragmatic observational study and consequently, we were unable to prescribe the timing and quantity of oxygen measurements that is suitable for an individual patient in ICU. We have clarified this in the limitations section. While we agree that might be variations in sampling episodes between patients and would have introduced precision bias, this is highly unlikely as the sampling episodes were similar between survivors and non-survivors (Table). Moreover, all the patients presented here were very sick and required mechanical ventilation, representing a unique homogenous cohort with similarities in their degree of hypoxia. We presented all available blood samples to minimise any selection bias. We have modified the sentence in the limitations to address this comment. “Our data is clearly susceptible to sampling bias, as the sickest patients tend to get more frequent blood gas sampling than more stable patients. However, our results show that those who died were not sampled excessively when compared with non-survivors”.*

Minor points:
2) P21, L353-357: Discussion about mitochondrial dysfunction looks too strong and too assertive, even though I agree with the opinion that mitochondrial function should affect oxygen utilization and clinical outcome of this patient population.

*Response: We have modified this sentence to reflect the reviewer’s comments. “There is increasing evidence that upon cell entry the SARS-CoV-2 virus hijacks host's mitochondria which may contribute to mitochondrial dysfunction and cellular death”.*

3) P22, L381-382: The same as above.

*Response: We have modified the sentence to reflect the reviewer’s comments. “Urgent further evaluation of the relationship between impaired oxygen extraction and survival in COVID-19 is justified”.*

The changes within the manuscript are highlighted in yellow. We are looking forward to hearing from you soon.

Thank you for considering this manuscript for publication in PLOS one.

Yours sincerely,

# Professor Mike Grocott

# BSc MBBS MD FRCA FRCP FFICM

Professor of Anaesthesia and Critical Care Medicine, Head, Integrative Physiology and Critical Illness Group, CES Lead, Critical Care Research Area, Southampton NIHR Respiratory BRC
